# The association of CCAT2 rs6983267 SNP with MYC expression and progression of uterine cervical cancer in the Polish population

**DOI:** 10.1007/s00404-018-4740-6

**Published:** 2018-03-10

**Authors:** Sebastian Łaźniak, Anna Lutkowska, Żaneta Wareńczak-Florczak, Anna Sowińska, Alexander Tsibulski, Andrzej Roszak, Stefan Sajdak, Pawel P. Jagodziński

**Affiliations:** 1 Department of Biochemistry and Molecular Biology, Poznań University of Medical Sciences, 6 Święcickiego St., 60-781 Poznan, Poland; 2 Department of Radiotherapy and Gynecological Oncology, Greater Poland Cancer Center, Pozna, Poland; 3 Department of Electroradiology, Poznań University of Medical Sciences, Poznan, Poland; 4 Department of Computer Science and Statistics, Poznań University of Medical Sciences, Poznan, Poland; 5 Clinic of Gynecological Surgery, Poznań University of Medical Sciences, Poznan, Poland

**Keywords:** Cervical squamous cell carcinoma, MYC, Polymorphism

## Abstract

**Purpose:**

Previous studies have reported a significant contribution of NC_000008.10:g.128413305 G>T (rs6983267) single-nucleotide polymorphism (SNP) in the MYC enhancer region to the susceptibility of various cancers. However, the role of rs6983267 SNP in cervical cancer (CC) development and progression has not been demonstrated to date. Therefore, we evaluated the role of rs6983267 SNP in MYC expression in cervical cancers and non-cancerous cervical tissues. In addition, we assessed the role of this SNP in the development and progression of CC.

**Methods:**

Using high-resolution melting analysis, we evaluated rs6983267 SNP frequency in women diagnosed with cervical squamous cell carcinoma (SCC) (*n* = 481) and controls (*n* = 502) in a Polish Caucasian population. Logistic regression analysis was employed to adjust for the effects of age, parity, oral contraceptive use, tobacco smoking, and menopausal status.

**Results:**

Dividing patients based on clinical characteristics demonstrated an association of the rs6983267 genotype with tumor stage III and grade of differentiation G2 and G3. The *p* trend value calculated for the rs6983267 SNP in patients with stage III was 0.0006. We also observed a significant contribution of rs6983267 SNP to tumor grade of differentiation G2 and G3. Additional contributors were oral contraceptive use, smoking, and postmenopausal age. We found statistically significant increase of MYC transcript levels in cervical SCC tissues from carriers of the GG vs. T/T (*p* < 0.00001), G/T vs. T/T (*p* = 0.0002), and in the non-cancerous cervical tissues from carriers of the GG vs. T/T (*p* = 0.00046).

**Conclusion:**

The rs6983267 SNP may contribute to the increased MYC expression as well as the spread and rapid growth of cervical SCC as compared to lower grade carcinomas.

**Electronic supplementary material:**

The online version of this article (10.1007/s00404-018-4740-6) contains supplementary material, which is available to authorized users.

## Introduction

Cervical cancer (CC) is fourth among the common malignancies, which affect women worldwide [1]. The cervix is composed of an ectocervix which is covered by stratified squamous epithelium, and an endocervical canal consisting of mucus-secreting columnar epithelium [1, 2]. The ectocervix is more predisposed to transformation and development of squamous cell carcinoma (SCC), while the endocervix is more predisposed to adenocarcinomas [1, 2]. Less common histological subtypes of CC include adenosquamous, small cell/neuroendocrine, serous papillary, and clear cell carcinomas of the cervix [1, 2]. Cervical SCC account for approximately 80% of invasive CC cases [1]. Virtually, all cases of CC result from infection with the oncogenic strains of human papillomavirus (HPV) [3, 4]. The majority of HPV infections are short-lived and resolve spontaneously [5–7]. However, in some individual cases, permanent infection will lead to development of a precancerous lesion, known as cervical intraepithelial neoplasia or adenocarcinoma in situ [5] Additional factors increasing the likelihood of the development of CC include tobacco smoking, long-term oral contraceptive use, high parity, early sexual intercourse, pollutants, and co-infection with type 2 herpes simplex virus or the human immunodeficiency virus [8–10].

Cervical carcinoma development displays strong genetic predisposition, especially with history of cancer in a first-degree relative [11–13] Genome-wide association studies (GWAS) demonstrated MHC loci as major genetic components in the development of CC [14–16]. Various previously studied polymorphisms failed to reach GWAS-statistical significance, but, nevertheless, they may still contribute to the progression of CC [17].

Recently, it has been reported that the integrated HPV fragment creates long-distance interactions with MYC gene and 8q24.22 region, therefore, increasing the allele-specific MYC expression in cervical cancer [18] HeLa cells. The previous studies demonstrated a significant contribution of NC_000008.10:g.128413305 G>T (rs6983267) single-nucleotide polymorphism (SNP) in the MYC enhancer region to the susceptibility to various cancers [19–25]. However, the role of rs6983267 SNP in CC development and progression has not been demonstrated to date. In our study, we assessed the prevalence of rs6983267 SNP in women with cervical SCC in the Polish Caucasian population. We also evaluated the distribution of rs6983267 in stages I–IV and the differentiation grades of cervical SCC.

## Methods

### Study population

**Fig. 1:**
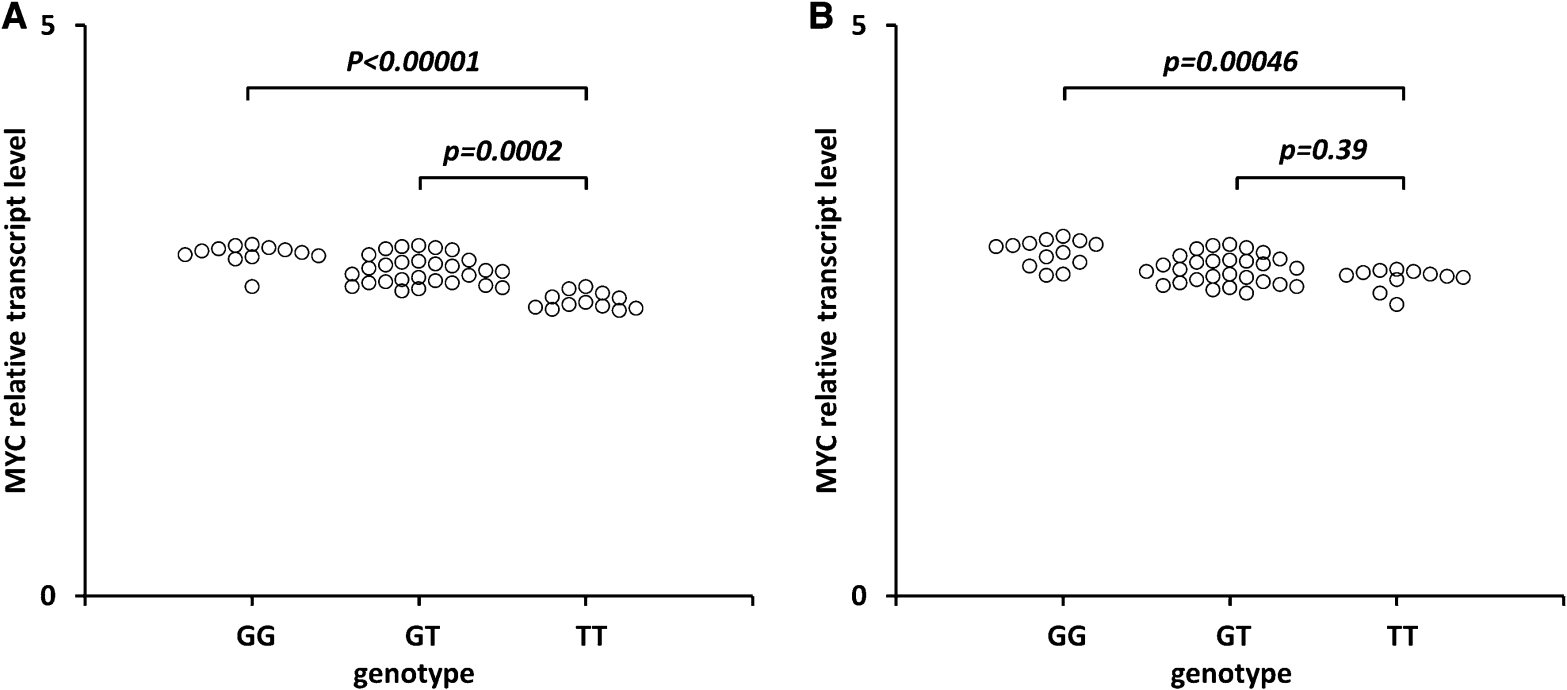
Effect of the rs6983267 SNP on MYC transcript levels in cancerous cervical tissues (**a**) and non-cancerous tissues (**b**). Frozen tissue was homogenized, followed by total RNA isolation. Quantitative analyses of MYC transcript levels were performed by qRT-PCR using the SYBR Green I system. The quantity of MYC transcript levels in each sample was standardized by the geometric mean of references using HMBS and B2M cDNA levels. Kruskal–Wallis test with ^a^Dunn’s post hoc

The studied subjects included 481 patients diagnosed with cervical SCC, with stage and grade of differentiation evaluated according to the International Federation of Gynecology and Obstetrics (FIGO) classification system and World Health Organization (Table 1). Patient data and primary cervical SCC tissue samples were obtained from subjects enrolled between March 2008 and December 2016 at the Department of Radiotherapy of the Greater Poland Cancer Center in Poznań, Poland (Fig. 1). The control group included 502 healthy females randomly selected during medical examinations at the Institute of Mother and Child, Warsaw (Table 1).

**Table 1:** Clinical and demographic characteristics of patients with cervical squamous cell carcinoma and controls

Characteristic	Patients (*n* = 481)	Controls (*n* = 502)	*p* ^b^
Mean age (years) ± SD^a^	51.4 ± 9.6	51.7 ± 9.1	
Tumor stage			
IA	62 (12.8%)		
IB	61 (12.7%)		
IIA	59 (12.3%)		
IIB	57 (11.9%)		
IIIA	152 (31.6%)		
IIIB	61 (12.7%)		
IVA	14 (2.9%)		
IVB	15 (3.1%)		
Histological grade			
G1	151 (31.4%)		
G2	101 (21.0%)		
G3	167 (34.7%)		
Gx	62 (12.9%)		
Parity			
Never	61 (12.68%)	55 (10.96%)	
Ever	420 (87.32%)	447 (89.04%)	0.402^b^
Oral contraceptive pill use			
Never	262 (54.47%)	289 (57.57%)	
Ever	219 (45.53%)	213 (42.43%)	0.328^b^
Tobacco smoking			
Never	308 (64.03%)	332 (66.14%)	
Ever	173 (35.97%)	170 (33.86%)	0.489^b^
Menopausal status			
Premenopausal	170 (35.34%)	194 (38.65%)	
Postmenopausal	311 (64.66%)	308 (61.35%)	0.284^b^
HPV genotypes			
16 and 18	343 (71.3%)		
16, 18, 31, 33, 35, 39, 45, 51, 52, 56, 58, 59 and 68	389 (80.9%)		

^a^Age at first diagnosis

^b^Chi-squared, *p* statistical *p* value

Information regarding the parity of at least one, oral contraceptive use, active tobacco smoking within the last 12 months, and menopausal status was obtained as part of the control and patient history.

### Tissue samples

The primary SCC tissue samples were obtained from 51 patients with mean age of 52.4 ± 9.6 years and classified as stage III at the time of surgery. The non-cancerous cervical tissue samples were obtained from 52 women with a mean age of 51.8 ± 9.7 years with uterine leiomyomas undergoing uterine surgical resection in the Clinic of Gynecological Surgery, University of Medical Sciences, Poznań, Poland. A portion of the tissue sample was immediately snap-frozen in liquid nitrogen and stored at − 80 °C until RNA isolation was performed.

### Study ethics

All the patients and controls were Polish Caucasians and written consent was obtained from all participating individuals. The study procedures were approved by the Local Ethical Committee of the Poznań University of Medical Sciences (reference number of ethical approval: 285/16 and 566/16).

### Genetic analysis

DNA was isolated from peripheral blood cells via a salting-out procedure. The primers were designated using Oligo 7.6 software (DBA Oligo, Inc., Colorado Springs, CO). The NC_000008.10:g.128413305 G>T polymorphism DNA fragment (170 bp) was amplified using the primers (forward 5′ TAACCTCTTCCTATCTCA 3′ and reverse 5′ AAATAAAGTCAATAGCACAT 3′). The rs6983267 SNP was then genotyped via high-resolution melting (HRM) curve analysis using HOT FIREPol EvaGreen (Solis BioDyne, Tartu, Estonia) with a LightCycler 480 system (Roche Diagnostics, Mannheim, Germany). The presence of this SNP was reanalyzed by Sanger sequencing analyses of arbitrarily chosen samples, comprising 10% of the samples from both cases and controls. The concordance rate between HRM and sequencing was 100%.

### Reverse transcription and quantitative real-time PCR (qRT-PCR) analysis of MYC transcript levels in cervical SCC and non-cancerous tissues

Frozen SCC and non-cancerous tissues were homogenized and total RNA was isolated according to the method of Chomczyński and Sacchi [26]. RNA quality was determined spectrophotometrically using a BioPhotometer^®^ from Eppendorf AG (Hamburg, Germany) and agarose gel electrophoresis. RNA samples were treated with DNase I, quantified, and reverse-transcribed (RT) into complimentary DNA (cDNA) using the Moloney Murine Leukemia Virus (M-MLV) from Invitrogen (Life Technologies, Carlsbad, CA) (Supplementary data 1).

Quantitative analysis of MYC cDNA isoforms (Supplementary data 1) was performed by Light Cycler^®^480 II Real-Time PCR System (Roche Diagnostics GmbH, Mannheim, Germany), using SYBR Green I as detection dye. MYC cDNA was quantified using the relative quantification method with a calibrator. The calibrator was prepared with a cDNA mix from all cDNA samples and consecutive dilutions were used to create a standard curve. For amplification, 1 μl of cDNA solution was added to 9 μl of LightCycler 480 SYBR Green I Master Mix (Roche Diagnostics GmbH, Mannheim, Germany) and primers (Supplementary data 1).

The quantity of MYC transcript in each sample was standardized by the geometric mean of reference transcript levels: hydroxymethylbilane synthase (HMBS) and beta-2-microglobulin (B2 M). The PCR amplification efficiency for target and reference cDNA was determined using different standard curves created by consecutive dilutions of the cDNA template mixture. The MYC cDNA, HMBS, and B2 M cDNA were amplified using the primer pairs presented in Supplementary data 1. The MYC mRNA levels were expressed as multiples of these cDNA concentrations in the calibrator.

### Statistical analysis

The distinction in genotypic prevalence between the patients and controls and their genotype deviation from Hardy–Weinberg (HW) equilibrium were evaluated using a *χ*^2^ test. The rs6983267 SNP was tested for association with cervical SCC using the Cochran–Armitage *p* trend test (*p*_trend_). The *χ*^2^ and Fisher’s exact tests were used to determine the differences in genotypic distributions between the patients and controls. The odds ratio (OR) and 95% confidence intervals (95% CI) were also calculated. A logistic regression analysis was used to adjust for the effect of confounders such as age, parity, oral contraceptive use, tobacco smoking, and menopausal status. A *p* value of < 0.05 was considered statistically significant. Statistical analysis of comparing MYC transcript levels between the G/G vs. T/T and T/G vs. T/T genotype carriers was evaluated using the Kruskal–Wallis test with Dunn’s post hoc. Statistical analyses were conducted using Statistica version 10, 2011 (Stat Soft, Inc., Tulsa, USA).

## Results

### Prevalence of the rs6983267 SNP among all women with cervical SCC and healthy women

The values for the Chi-square (*χ*^2^) test of HW equilibrium were 0.119 and 0.188 for the patients and controls, respectively. The statistical evaluation of the rs6983267 genotype distribution in women with SCC and healthy women is stated in Table 2. We did not find a significant association of rs6983267 SNP with all patients with cervical SCC, the *p* trend value calculated for the rs6983267 polymorphism was *p*_trend_ = 0.146. The logistic regression analysis, which adjusted for the effects of age, parity, oral contraceptive use, tobacco smoking, and menopausal status, also did not demonstrate an association of rs6983267 SNP with cervical SCC (Table 2). For G/G vs. T/T adjusted OR was 1.139 (95% CI 0.906–1.432, *p* = 0.262), for T/G vs. T/T adjusted OR was 1.505 (95% CI 1.059–2.137, *p* = 0.056), and for T/G + G/G vs. T/T adjusted OR was 1.307 (95% CI 0.938–1.822, *p* = 0.113).

**Table 2:** Prevalence of the rs6983267 polymorphism among patients with SCC and controls

Genotype	Patients (frequency)	Controls (frequency)	Odds ratio (95% CI)	*p* ^a^	Adjusted odds ratio (95% CI)^c^	*p*	*p*_trend_
All							
T/T	104 (0.21)	126 (0.25)	Referent		Referent		0.146
T/G	263 (0.55)	271 (0.54)	1.176 (0.862–1.603)	0.306	1.505 (1.059–2.137)	0.056	
G/G	114 (0.24)	105 (0.21)	1.315 (0.908–1.907)	0.147	1.139 (0.906–1.432)	0.262	
G/T + G/G	377 (0.78)	376 (0.75)	1.215 (0.9031–1.634)	0.198	1.307 (0.938–1.822)	0.113	
MAF^d^	0.51	0.48					
Tumor stage							
IA + IB							
T/T	37 (0.30)	126 (0.25)	Referent	–	Referent	–	0.417
T/G	61 (0.50)	271 (0.54)	0.767 (0.484–1.214)	0.256	1.395 (0.797–2.440)	0.243	
G/G	25 (0.20)	105 (0.21)	0.811 (0.459–1.433)	0.470	1.045 (0.739–1.479)	0.801	
G/T + G/G	86 (0.70)	376 (0.75)	0.779 (0.504–1.203)	0.259	1.180 (0.699–1.991)	0.535	
MAF^d^	0.50	0.48					
IIA + IIB							
T/T	33 (0.28)	126 (0.25)	Referent	–	Referent	–	0.377
T/G	62 (0.53)	271 (0.54)	0.874 (0.545–1.401)	0.575	0.712 (0.346–1.081)	0.090	
G/G	21 (0.18)	105 (0.21)	0.764 (0.417-1.399)	0.382	0.863 (0.456–0.964)	0.081	
G/T + G/G	83 (0.72)	376 (0.75)	0.8428 (0.5368–1.323)	0.4571	0.670 (0.274–0.806)	0.096	
MAF^d^	0.45	0.48					
IIIA + IIIB							
T/T	30 (0.14)	126 (0.25)	Referent	–	Referent	–	0.0006
T/G	122 (0.57)	271 (0.54)	**1.891 (1.203–2.971)**	**0.0053**	**2.604 (1.539–4.406)**	**0.0003**	
G/G	61 (0.29)	105 (0.21)	**2.440 (1.468-4.056)**	**0.0005**	**1.659 (1.567–2.381)**	**0.006**	
G/T + G/G	183 (0.86)	376 (0.75)	**2.044 (2.322–3.160)**	**0.0011**	**2.356 (1.453–3.820)**	**0.0005**	
MAF^d^	0.57	0.48					
IVA + IVB							
T/T	4 (0.14)	126 (0.25)	Referent	–	Referent		0.2592
T/G	18 (0.62)	271 (0.54)	2.092 (0.694–6.311)	0.239^b^	NA		
G/G	7 (0.24)	105 (0.21)	2.100 (0.598–7.372)	0.354^b^	NA		
G/T + G/G	25 (0.86)	376 (0.75)	2.094 (0.715–6.136)	0.191^b^	NA		
MAF^d^	0.55	0.48					
Differentiation grade							
G1							
T/T	37 (0.24)	126 (0.25)	Referent	–	Referent	–	0.509
T/G	77 (0.51)	271 (0.54)	0.968 (0.620–1.511)	0.885	0.590 (0.343–1.014)	0.056	
G/G	37 (0.25)	105 (0.21)	1.200 (0.710- 2.027)	0.495	0.873 (0.633–1.204)	0.407	
G/T + G/G	114 (0.75)	376 (0.75)	1.032 (0.6769–1.575)	0.882	0.527 (0.315–0.881)	0.074	
MAF^d^	0.50	0.48					
G2							
T/T	27 (0.27)	126 (0.25)	Referent	–	Referent	–	0.8123
T/G	53 (0.52)	271 (0.54)	0.913 (0.548–1.519)	0.725	**2.798 (1.398–5.597)**	**0.004**	
G/G	21 (0.21)	105 (0.21)	0.933 (0.499–1.746)	0.829	1.257 (0.836–1.889)	0.269	
G/T + G/G	74 (0.73)	376 (0.75)	0.9184 (0.5656–1.491)	0.731	**2.058 (1.090–3.885)**	**0.026**	
MAF^d^	0.47	0.48					
G3							
T/T	24 (0.14)	126 (0.25)	Referent	–	Referent	–	0.0068
T/G	99 (0.59)	271 (0.54)	**1.918 (1.171- 3.142)**	**0.0089**	**3.032 (1.668–5.510)**	**0.0003**	
G/G	44 (0.26)	105 (0.21)	**2.200 (1.256–3.855)**	**0.0053**	1.385 (0.925–2.074)	0.113	
G/T + G/G	143 (0.86)	376 (0.75)	**1.997 (1.239–3.218)**	**0.0040**	**2.330 (1.365–3.978)**	**0.002**	
MAF^d^	0.56	0.48					
Gx							
T/T	16 (0.26)	126 (0.25)	Referent	–	Referent	–	0.8032
T/G	34 (0.55)	271 (0.54)	0.988 (0.526-1.857)	0.970	0.988 (0.456–2.138)	0.975	
G/G	12 (0.19)	105 (0.21)	0.900 (0.408- 1.987)	0.795	0.939 (0.595–1.483)	0.788	
G/T + G/G	36 (0.58)	376 (0.75)	0.754 (0.405–1.405)	0.373	1.079 (0.537–2.169)	0.831	
MAF^d^	0.47	0.48					

Significant results are highlighted in bold font. NA—the number of genotypes is too small, and therefore, the logistic regression does not apply

^a^*χ*^2^ or ^b^Fisher’s exact test

^c^ORs were adjusted by age, parity, oral contraceptive use, tobacco smoking, and menopausal status

^d^Minor allele frequency

### Prevalence on of the rs6983267 SNP among cervical SCC women for different tumor stages and grade of differentiation.

Stratification of patients based on clinical characteristics demonstrated an association of the rs6983267 genotype with tumor stages III and grade of differentiation G2 and G3 (Table 2). The *p* trend value calculated for the rs6983267 SNP in cervical SCC patients with stage III was statistically significant (*p*_trend_ = 0.0006). Adjusting for the effect of age, parity, oral contraceptive use, tobacco smoking, and menopausal status in patients with stage III, the logistic regression analysis revealed that the G/G vs. T/T genotype may be a risk factor of cervical SCC with an adjusted OR 1.659 (95% CI 1.567–2.381, *p* = 0.006). There was also significant association with SCC for the T/G vs. T/T genotype, with an adjusted OR 2.604 (95% CI 1.539–4.406, *p* = 0.0003) and for the T/G + G/G vs. T/T genotype, with an adjusted OR 2.356 (95% CI 1.453–3.820, *p* = 0.0005) in stage III patients. In women with differentiation grade G2, the *p* trend value calculated for rs6983267 SNP was not statistically significant (*p*_trend_ = 0.812). There was also no risk effect of the G/G vs. T/T genotype with an adjusted OR 1.257 (95% CI 0.836–1.889, *p* = 0.269). However, we found a risk effect of the T/G vs. T/T genotype with an adjusted OR 2.798 (95% CI 1.398–5.597, *p* = 0.004) and for T/G + G/G vs. T/T with an adjusted OR 2.058 (95% CI 1.090–3.885, *p* = 0.026).

In patients with grade of differentiation G3, the *p* trend value calculated for the rs6983267 SNP was statistically significant (*p*_trend_ = 0.0068). We found a risk effect of the T/G vs. G/G genotype, with an adjusted OR 3.032 (95% CI 1.668–5.510, *p* = 0.0003) and for T/G + G/G vs. T/T with an adjusted OR 2.330 (95% CI 1.365–3.978, *p* = 0.002). However, we did not observe significant association for G/G vs. T/T genotype, with an adjusted OR 2.330 (95% CI 1.365–3.978, *p* = 0.002). The logistic regression analysis did not show any association of the rs6983267 SNP with tumor stage I and IV and grade of differentiation G1 and GX (Table 2). Moreover, there was no association of the rs6983267 SNP with HPV strains to neither SCC nor tumor stages I, II, III, and IV and grades of differentiation G1, G2, G3, and GX (data not shown).

### Distribution of the rs6983267 SNP among women with SCC and healthy women with history of parity, oral contraceptive use, tobacco smoking, or menopausal status

Stratification of patients for rs6983267 SNP demonstrated an association of this polymorphism with positive history of oral contraceptive use, smoking, and postmenopausal age (Table 3). The age-adjusted OR for women with a positive history of oral contraceptive use for G/G vs. T/T was 1.558 (95% CI 1.135–2.139, *p* = 0.0057); and for T/G + G/G vs. T/T, the age-adjusted OR was 1.687 (95% CI 1.048–2.715, *p* = 0.0309). The age-adjusted OR for women with a history of tobacco smoking for T/G vs. T/T was 2.098 (95% CI 1.248–3.527, 0.005). In women of postmenopausal age, we found contribution of rs6983267 SNP to the risk of cervical SCC. The age-adjusted OR for postmenopausal women for T/G vs. T/T was 2.362 (95% CI 1.466–3.804, *p* = 0.004); for G/G vs. T/T adjusted OR was 1.797 (95% CI 1.379–2.341, *p* = 0.0001); and for T/G + G/G vs. T/T adjusted OR was 2.724 (95% CI 1.723–4.307, *p* = 0.0002).

**Table 3:** Distribution of rs6983267 genotypes among SCC risks: parity, oral contraceptive use, tobacco smoking, and menopausal status

High-risk exposure	Patients		Controls		Adjusted odds ratio (95% CI)	*p*	Adjusted odds ratio (95% CI)	*p*
Genotype	Ever	Never	Ever	Never	Ever		Never	
Parity								
T/T	64	40	85	41	Referent		Referent	
T/G	242	21	261	10	1.212 (0.837–1.756)	0.308	2.185 (0.903–5.289)	0.0797
G/G	114	0	101	4	1.377 (0.894–2.120)	0.1452	NA	0.999
T/G + G/G	356	21	362	14	1.307 (0.915–1.866)	0.1410	1.572 (0.693–3.565)	0.273
Oral contraceptive use								
T/T	36	68	53	73	Referent		Referent	
T/G	131	132	130	141	1.530 (0.935–2.506)	0.0969	0.954 (0.631–1.443)	0.824
G/G	52	62	30	75	**1.558 (1.135**–**2.139)**	**0.0057**	0.926 (0.723–1.186)	0.539
T/G + G/G	183	194	160	**216**	**1.687 (1.048**–**2.715)**	**0.0309**	0.958 (0.651–1.409)	0.827
Smoking								
T/T	47	57	52	74	Referent		Referent	
T/G	106	157	57	214	**2.098 (1.248**–**3.527)**	**0.0050**	0.945 (0.631–1.414)	0.781
G/G	20	94	61	44	0.599 (0.433–0.828)	0.0618	1.580 (1.218–2.049)	0.065
T/G + G/G	126	251	118	258	1.144 (0.713–1.836)	0.575	1.263 (0.856–1.862)	0.238

	Premenopausal	Postmenopausal	Premenopausal	Postmenopausal	Premenopausal		Postmenopausal	
Menopausal status								
T/T	63	41	55	71	Referent		Referent	
T/G	92	171	104	167	0.663 (0.422–1.042)	0.183	**2.362 (1.466**–**3.804)**	**0.004**
G/G	15	99	35	70	0.339 (0.204–0.563)	0.0723	**1.797 (1.379**–**2.341)**	**0.0001**
T/G + G/G	97	280	139	237	0.620 (0.335–0.906)	0.0821	**2.724 (1.723**–**4.307)**	**0.0002**

All *p* values were adjusted by age. Significant results are highlighted in bold font. NA—the number of genotypes is too small, and therefore, the logistic regression does not apply

### The rs6983267 SNP is associated with increased MYC transcript levels in cervical SCC and non-cancerous tissues

We found statistically significant increase of MYC transcript levels in cervical SCC tissues from carriers of the GG vs. T/T (*p* < 0.00001) and G/T vs. T/T (*p* = 0.0002) (Fig. 1a). We also found a statistically significant increase of the MYC transcript levels in the non-cancerous cervical tissues from carriers of the GG vs. T/T (*p* = 0.00046) but not for G/T vs. T/T (*p* = 0.39) (Fig. 1b).

## Discussion

Recent genome-wide association studies (GWASs) have identified rs6983267 in chromosome 8q24 as a new susceptibility locus for several cancers, including breast, prostate, and colorectal cancer [19–21]. It has also been reported that rs6983267 may contribute to the susceptibility to ovarian cancer among premenopausal Chinese women, as well as endometrial and thyroid cancers [22–24]. The rs6983267 SNP has also been significantly associated with susceptibility to lung cancer or platinum-based chemotherapy response [25]. The function of rs6983267 SNP remained elusive, because it was mapped in a gene desert which does not contain any protein encoding genes. The rs6983267 SNP is situated 335 kb downstream of the *MYC* proto-oncogene [27, 28]. Several studies have demonstrated a disputable association between rs6983267 and proto-oncogene MYC expression [29–31]. The *MYC* oncogene is a target gene of the Wnt signaling pathway, which is constitutively activated in the early development of various cancers including CC [32].

In our study, we found an association of rs6983267 SNP with stages III and grade of differentiation G2 and G3. Our observations suggest that the G variant of rs6983267 SNP induces the spread of cervical SCC cells to the surrounding tissues and promotes rapid growth as compared to lower grade tumor cells. Moreover, we observed significantly upregulated MYC transcript levels in G/G as compared to T/T carriers in non-cancerous tissues and in carriers of the G allele as compared to carriers of the T/T genotype in the cervical SCC tissues.

Ling et al. [36] demonstrated that long non-coding RNAs (lncRNAs) of Colon Cancer-Associated Transcript 2 (CCAT2), a transcript encompassing the rs6983267 SNP, up-regulates *MYC* through TCF7L2-mediated transcription. This results in the risk of allele G of rs6983267 to produce more lncRNAs CCAT2 transcript [33]. Recently, Redis et al. [37] reported that lncRNAs CCAT2 regulates cancer metabolism in vitro and in vivo in an allele-specific manner by binding the Cleavage Factor I (CFIm) complex with distinct affinities for the two subunits CFIm25 and CFIm68 [34]. The 8q24 region also contains lncRNAs CARLo-5, the expression of which has been increased in gastric, colon, lung, and endometrial cancers, where CARLo-5 is used as a modular scaffold [23, 35–38]. The oncogenic rs6983267 polymorphism interacts through long-range mechanism with promoter control *CARLo*-*5* transcription [29]. The lncRNAs are involved in many cellular processes within cancer biology and display tumor suppressive and oncogenic functions in various types of cancer [39].

Moreover, we found an association of increased risk of cervical SCC with rs6983267 SNP in patients with positive history of oral contraceptive use, tobacco smoking, and in women of postmenopausal age. This is in agreement with the previous studies suggesting the causative influence of oral contraceptive use, tobacco smoking, and postmenopausal age in cervical cancer development [8–10].

Our study is the first to demonstrate rs6983267 SNP as a risk factor of cervical carcinogenesis in Caucasian Polish individuals with positive history of oral contraceptive use, tobacco smoking, and postmenopausal age. We also observed that the rs6983267 G variant was associated with increased MYC transcript levels and enhanced growth and spread of cancer cells to neighboring tissues. However, this study should be repeated in other independent cohorts.

## Electronic supplementary material

Below is the link to the electronic supplementary material.

Supplementary material 1 (DOCX 29 kb)
