# Supplementary material for: The association of CCAT2 rs6983267 SNP with MYC expression and progression of uterine cervical cancer in the Polish population
Source: Arch Gynecol Obstet. 2018 Mar 10;297(5):1285–92. doi: 10.1007/s00404-018-4740-6 (PMC5897468; doi:10.1007/s00404-018-4740-6)
Supplement: Supplementary file 1 — Supplementary material 1 (DOCX 29 kb) [file 404_2018_4740_MOESM1_ESM.docx]

**Suplementarny data 1**

| **Item to check** | **Importance** |  |
| --- | --- | --- |
| **Experimental design** | | |
| Definition of experimental and control groups | E | The primary SCC tissue samples were obtained from 51 patients with stage III at the time of surgery. The non-cancerous cervical tissue samples were obtained from 52 women with uterine leiomyomas undergoing uterine surgical resection. Both primary and control group include patients with GG, GT and TT genotype. |
| Number within each group | E | SCC includes 12 patients with GG, 28 patients with GT, and 11 patients with TT genotype. Non-cancerous tissues group includes 13 patients with GG, 28 patients with GT, and 11 patients with TT genotype |
| Assay carried out by the core or investigator’s laboratory? | D | **N/A** |
| Acknowledgment of authors’ contributions | D | Agnieszka Mikuczewska |
| Sample | | |
| Description | E | SCC and non-cancerous tissues |
| Volume/mass of sample processed | D |  |
| Microdissection or macrodissection | E | N/A |
| Processing procedure | E | N/A |
| If frozen, how and how quickly? | E | Frozen -80°C |
| If fixed, with what and how quickly? | E | N/A |
| Sample storage conditions and duration (especially for FFPE^b^ samples) | E | -80°C |
| Nucleic acid extraction | | |
| Procedure and/or instrumentation | E | Chomczyński and Sacchi method |
| Name of kit and details of any modifications | E | N/A |
| Source of additional reagents used | D |  |
| Details of DNase or RNase treatment | E | RNA samples were treated with DNase I |
| Contamination assessment (DNA or RNA) | E | qPCR amplification of genomic DNA fragment |
| Nucleic acid quantification | E | spectrophotometrically |
| Instrument and method | E | BioPhotometer® Eppendorf AG (Hamburg, Germany) |
| Purity (A260/A280) | D |  |
| Yield | D |  |
| RNA integrity: method/instrument | E | agarose gel electrophoresis |
| RIN/RQI or Cq of 3_ and 5_ transcripts | E | N/A |
| Electrophoresis traces | D |  |
| Inhibition testing (Cq dilutions, spike, or other) | E | Cq dilutions of cDNA |
| Reverse transcription | | |
| Complete reaction conditions | E | 1ug of Total RNA was dissolved in 8.25 ul H_2_O and added to mix (Total volume 5ul) composed of 0.5ul oligo dT (final concentration 2.5uM), hexamers (final concentration 2.5uM) and 4ul dNTP (final concentration 2.5uM) followed by 5 minute incubation in 70^o^C. After that reaction mixture was kept 1 minute on ice. In the second step the reaction mixture composed of 4 ul M-MLV buffer (final concentration x1), 2ul DTT (final concentration 10mM), 1ul RNase OUT (final conc. 40U/ul) and 0.5ul M-MLV (final conc. 100U/ul) was added to the first solution and then incubated 10 min in 25 ^o^C, 1 hour in 37 ^o^C, and 15 min in 75 ^o^C |
| Amount of RNA and reaction volume | E | 1 μg of RNA, reaction volume 20 μl |
| Priming oligonucleotide (if using GSP) and concentration | E | oligo d(T) (final conc. 2.5uM) + hexamers (final conc. 2.5uM) |
| Reverse transcriptase and concentration | E | Moloney Murine Leukemia Virus (M-MLV) (final conc. 40U/ul ) |
| Temperature and time | E | according to the manufacturer’s protocol |
| Manufacturer of reagents and catalogue numbers | D | Invitrogen, Life Technologies, (Carlsbad, CA) , 28025013 |
| Cq^s^ with and without reverse transcription | D^c^ |  |
| Storage conditions of cDNA | D | -20°C |
| qPCR target information | | |
| Gene symbol | E | MYC |
| Sequence accession number (ENST) | E | ENST00000621592.5 |
| Location of amplicon | D | Position 1286-1377 of MYC transcript (2366 nt) |
| Amplicon length | E | 92 bp |
| In silico specificity screen (BLAST, and so on) | E | BLASTN 2.5.1+ (https://blast.ncbi.nlm.nih.gov/) |
| Pseudogenes, retropseudogenes, or other homologs? | D |  |
| Sequence alignment | D | BLASTN 2.5.1+ (https://blast.ncbi.nlm.nih.gov/) |
| Secondary structure analysis of amplicon | D | Oligo 7.6 software (http://www.oligo.net/downloads.html) |
| Location of each primer by exon or intron (if applicable) | E | Forward primer – exon 2  Reverse primer – exon 3 |
| What splice variants are targeted? | E | MYC-207 (Reference Sequence) |
| qPCR oligonucleotides | | |
| Primer sequences | E | MYC-forward: TGCTCCATGAGGAGACACC  MYC-reverse: CTTTTCCACAGAAACAACATCG  PBGD-forward: GCCAAGGACCAGGACATC  PBGD-reverse: TCAGGTACAGTTGCCCATC  B2M-forward: CACCCCCACTGAAAAAGATG  B2M-reverse: CCTCCATGATGCTGCTTACA |
| RTPrimerDB identification number | D | N/A |
| Probe sequences | D^d^ | N/A |
| Location and identity of any modifications | E | N/A |
| Manufacturer of oligonucleotides | D | Oligo.pl (Institute of Biochemistry and Biophysics Polish Academy of Sciences, Warsaw, Poland, http://oligo.ibb.waw.pl/) |
| Purification method | D | Salting-out method with ethanol precipitation |
| qPCR protocol | | |
| Complete reaction conditions | E | 5 μl Master Mix (2×conc.) + 1 μl MgCl_2_ (25mM) + 1 μl F and R primer mix (5 μM each) + 1 μl cDNA + 2 μl H_2_O |
| Reaction volume and amount of cDNA/DNA | E | Reaction volume 10 μl (cDNA 1 μl) |
| Primer, (probe), Mg^2+^, and dNTP concentrations | E | Primer 0.5 μM, Mg^2+^ 2.5 mM, dNTP 0.8 mM |
| Polymerase identity and concentration | E | LightCycler 480 SYBR Green I Master Mix (Roche Diagnostics GmbH, Mannheim, Germany) |
| Buffer/kit identity and manufacturer | E | LightCycler 480 SYBR Green I Master Mix (Roche Diagnostics GmbH, Mannheim, Germany) |
| Exact chemical composition of the buffer | D | N/A |
| Additives (SYBR Green I, DMSO, and so forth) | E | SYBR Green I |
| Manufacturer of plates/tubes and catalog number | D | Roche Diagnostics GmbH (Mannheim, Germany) |
| Complete thermocycling parameters | E | Preincubation: 10min 95°C,  Amplification: 10s 95°C, 10s 60°C, 10s 72°C with single fluorescence acquisition, 45 cycles, Ramp rate: 2.2°C (cooling) and 4.4°C (heating) Melting: 1min 95°C, 1min 40°C, 75-95°C with continuous fluorescence acquisition |
| Reaction setup (manual/robotic) | D | Manual |
| Manufacturer of qPCR instrument | E | Light Cycler®480 II Real-Time PCR System (Roche Diagnostics GmbH, Mannheim, Germany) |
| qPCR validation | | |
| Evidence of optimization (from gradients) | D | PCR with gradient temperature of annealing and electrophoresis |
| Specificity (gel, sequence, melt, or digest) | E | Melting curve |
| For SYBR Green I, Cq of the NTC | E | NTC with no amplification observed (Cq>40) |
| Calibration curves with slope and y intercept | E | The PCR amplification efficiency for target and reference cDNA was determined by different standard curves created by consecutive dilutions of the cDNA template mixture. |
| PCR efficiency calculated from slope | E | MYC E=1.0 (100%) |
| CIs for PCR efficiency or SE | D |  |
| r2 of calibration curve | E | R^2^ range from 0.99 to 1.0 |
| Linear dynamic range | E | cDNA dilution from 1 to 1/64 |
| Cq variation at LOD | E | N/A |
| CIs throughout range | D |  |
| Evidence for LOD | E | N/A |
| If multiplex, efficiency and LOD of each assay | E | N/A |
| Data analysis | | |
| qPCR analysis program (source, version) | E | Light Cycler®480 Software release 1.5.0 SP3 (Roche Diagnostics GmbH, Mannheim, Germany) |
| Method of Cq determination | E | Second derivative maximum method |
| Outlier identification and disposition | E | Peirce's criterion |
| Results for NTCs | E | No amplification observed for NTCs |
| Justification of number and choice of reference genes | E | Two reference genes: porphobilinogen deaminase (PBGD) and beta-2-microglobulin (B2M) |
| Description of normalization method | E | Relative quantification method with a calibrator. The calibrator was prepared as a cDNA mix from all cDNA samples and consecutive dilutions were used to create a standard curve. The quantity of MYC transcript in each sample was standardized by the geometric mean of transcript levels. |
| Number and concordance of biological replicates | D |  |
| Number and stage (reverse transcription or qPCR) of technical replicates | E | Three |
| Repeatability (intraassay variation) | E | N/A |
| Reproducibility (interassay variation, CV) | D | N/A |
| Power analysis | D | N/A |
| Statistical methods for results significance | E | the Kruskal-Wallis test with Dunn's post-hoc |
| Software (source, version) | E | Statistica version 10, 2011 (Stat Soft, Inc., Tulsa, USA) |
| Cq or raw data submission with RDML | D | N/A |

^a^All essential information (E) must be submitted with the manuscript. Desirable information (D) should be submitted if available. If primers are from RTPrimerDB, information on qPCR target, oligonucleotides, protocols, and validation is available from that source.

^b^ FFPE, formalin-fixed, paraffin-embedded; RIN, RNA integrity number; RQI, RNA quality indicator; GSP, gene-specific priming; dNTP, deoxynucleoside triphosphate.

^c^Assessing the absence of DNA with a no–reverse transcription assay is essential when first extracting RNA. Once the sample has been validated as DNA free, inclusion of a no–reverse transcription control is desirable but no longer essential.

^d^Disclosure of the probe sequence is highly desirable and strongly encouraged; however, because not all vendors of commercial predesigned assays provide this information, it cannot be an essential requirement. Use of such assays is discouraged.
